# Deciphering the gut microbiota's role in infection risk: Mendelian randomization analysis of microbial taxa and infectious outcomes

**DOI:** 10.3389/fmicb.2026.1821422

**Published:** 2026-09-21

**Authors:** Fengning Chen, Chi Zhang, Guangyue Yao, Ruiping Meng, Pengfei Fan, Leyan Chen, Yuxin Ou, Yan Jin

**Affiliations:** 1 Department of Clinical Laboratory, Shandong Provincial Hospital Affiliated to Shandong First Medical University, Jinan, China; 2 School of Public Health and Health Management, Shandong First Medical University, Jinan, Shandong, China; 3 Department of Hematology, Qilu Hospital of Shandong University, Cheeloo College of Medicine, Shandong University, Jinan, China; 4 School of Radiology, Shandong First Medical University, Tai'an, Shandong, China; 5 Department of Cardiology, Shandong Provincial Hospital Affiliated to Shandong First Medical University, Jinan, China; 6 School of Clinical and Basic Medicine, Shandong First Medical University, Jinan, China

**Keywords:** *Collinsella aerofaciens*, gut microbiota, infection, infectious diseases, Mendelian randomization

## Abstract

**Background:**

Observational studies have linked the gut microbiota (GM) to infectious diseases, but confounding, reverse causation, and disease-related microbiome changes limit causal interpretation. We combined Mendelian randomization (MR) with focused experimental analyses to investigate microbiota–infection relationships.

**Methods:**

Two-sample MR evaluated genetically predicted microbial taxa across 75 infectious outcomes. Inverse-variance weighted (IVW) estimates were used as the primary analysis, with weighted-median and MR-Egger estimates as complementary methods. Benjamini–Hochberg false discovery rate (FDR) and Bonferroni corrections were applied across all IVW tests. Instrument strength, Cochran's Q, MR-Egger intercepts, leave-one-out analysis, MR-PRESSO, and Steiger directionality testing were used to assess robustness. Exploratory association patterns were used to prioritize a Coriobacteriia-related respiratory signal for experimental investigation using *Collinsella aerofaciens* cell-free supernatant and A549 epithelial cells.

**Results:**

Among 15,728 IVW tests, 659 associations had nominal *P* < 0.05. After global correction, three taxonomically related associations with urinary tract infection (UTI) remained significant. Higher genetically predicted abundance of genus *Bifidobacterium* was associated with lower UTI risk (odds ratio [OR] 0.29, 95% confidence interval [CI] 0.17–0.49; FDR-adjusted *P* = 0.029), while order Bifidobacteriales and family Bifidobacteriaceae showed similar estimates (both OR 0.25, 95% CI 0.14–0.45) and met the Bonferroni threshold. Sensitivity analyses showed no significant heterogeneity or MR-PRESSO-detected outliers, and leave-one-out and Steiger analyses were supportive, although MR-Egger intercepts suggested possible directional pleiotropy. Exploratory analyses identified clustering between Coriobacteriia-lineage taxa and respiratory outcomes. *C. aerofaciens* cell-free supernatant altered 993 genes and enriched inflammatory pathways in A549 cells. qPCR supported increased CSF2, CXCL8, and CCL20 expression, while ELISA detected IL-8 and MIP-3α but not quantifiable GM-CSF. Untargeted metabolomics identified 201 differential metabolites between bacterial supernatant samples and matched BHI controls.

**Conclusion:**

The corrected *Bifidobacterium*-related UTI association was the principal MR finding, while the broader analysis identified exploratory microbiota–infection patterns. The *C. aerofaciens* model linked extracellular metabolic remodeling during bacterial growth with epithelial transcriptional and inflammatory responses, providing focused directions for further investigation.

## Introduction

Infectious diseases caused by viral, bacterial, fungal, and parasitic pathogens remain a major global health burden. For example, chronic hepatitis B virus infection affects approximately 254 million people worldwide (Jesudason, 2024; Li et al., 2025; WHO, 2026). The increasing prevalence of antimicrobial resistance has further complicated infection control and prompted the World Health Organization to prioritize clinically important drug-resistant pathogens (Jesudason, 2024; Nair et al., 2025). These challenges have stimulated interest in complementary approaches, including modulation of the gut microbiota (GM), to improve host resistance and reduce infection susceptibility (Ghani et al., 2022; Fishbein et al., 2023).

The GM contributes to epithelial barrier integrity, immune development, colonization resistance, and systemic immune homeostasis (Kulkarni and Newberry, 2025). Microbiome-directed approaches have also been proposed to preserve mucosal integrity and regulate inflammatory responses in conditions involving epithelial injury, including chemotherapy-induced mucositis in patients with breast cancer (Obeagu, 2026). Growing evidence also supports communication between the gut microbiota and distant organs through the gut–lung, gut–liver, gut–brain, and other microbiota-associated axes. Experimental studies have shown that germ-free animals exhibit impaired responses to acute respiratory infection and increased susceptibility to allergic airway disease (Fagundes et al., 2012; Olszak et al., 2012), while gut microbial signals also influence host immunity during influenza virus infection (Zhang et al., 2020; Liao et al., 2024; Rosshart et al., 2017). However, infection, antibiotic treatment, hospitalization, inflammation, and dietary changes can themselves alter microbial composition. Consequently, observational studies cannot readily determine whether microbiome alterations precede infection or arise as a consequence of disease and its treatment (Budden et al., 2017).

Mendelian randomization (MR) uses genetic variants as instrumental variables to evaluate potential causal relationships while reducing some forms of confounding and reverse causation (Davies et al., 2018). Previous MR studies have reported microbial associations with urinary tract infection (He et al., 2024), bidirectional relationships between microbial taxa and *Shigella* infection (Peng et al., 2024) and associations of selected bacterial species with influenza susceptibility or severity (Liao et al., 2024). However, most previous studies have focused on individual infections or restricted outcome groups. A systematic assessment of genetically predicted microbial taxa across diverse infectious outcomes, with global multiple-testing correction, instrument-strength evaluation, and complementary sensitivity analyses, remains limited.

We therefore evaluated genetically predicted microbial taxa across 75 infectious outcomes within a common two-sample MR framework. We hypothesized that microbial associations with infection susceptibility would be heterogeneous and that only a subset would remain supported after global multiple-testing correction and sensitivity analysis, whereas the broader nominal association landscape could help prioritize taxon–infection patterns for further investigation. Guided by the observed clustering between Coriobacteriia-lineage taxa and respiratory outcomes, we selected *Collinsella aerofaciens* as a representative experimental species. We subsequently examined epithelial transcriptional and inflammatory responses, together with extracellular metabolomic changes in the bacterial supernatant using RNA sequencing, untargeted metabolomics, qPCR, and ELISA.

## Results

## Multiple-testing correction prioritized *Bifidobacterium*-related associations with urinary tract infection

The primary screen comprised 15,728 IVW tests across microbial features and 75 infectious outcomes. A total of 659 exposure-outcome pairs had nominal *P* < 0.05. However, global correction substantially reduced the number of supported associations. Three taxonomically related results involving urinary tract infection (UTI) remained significant after correction (Figures 1A, B and Supplementary Table 1). Genetically predicted abundance of genus *Bifidobacterium* was associated with a lower risk of UTI (22 SNPs; OR 0.289, 95% CI 0.169–0.494; *P* = 5.56 x 10^−6^; FDR-adjusted *P* = 0.029). Order Bifidobacteriales and family Bifidobacteriaceae yielded identical protective estimates (19 SNPs for each; OR 0.253, 95% CI 0.144–0.445; *P* = 1.87 x 10^−6^; FDR-adjusted *P* = 0.0147), and both met the global Bonferroni threshold of 3.179 x 10^−6^.

**Figure 1:**
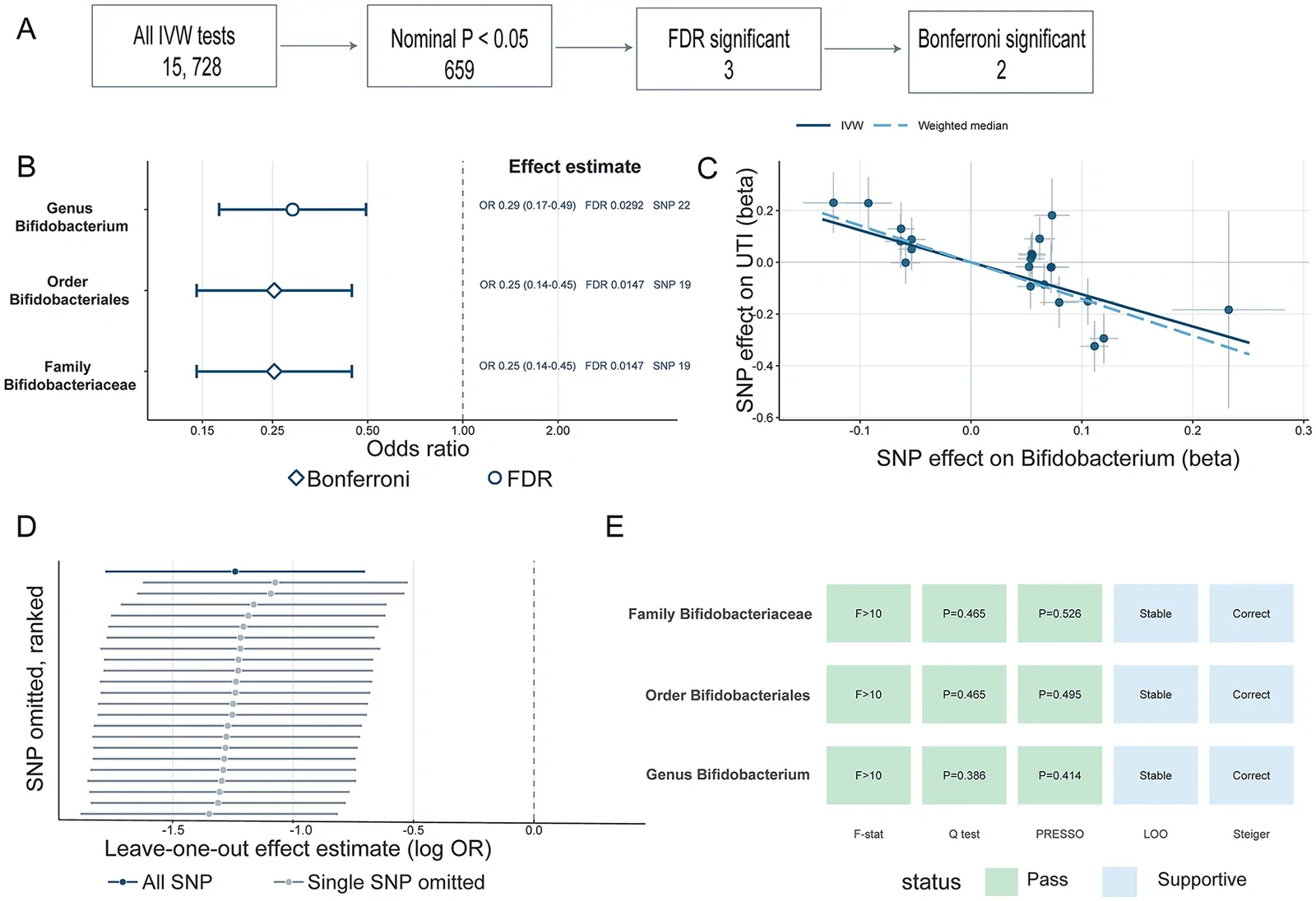
Corrected main MR findings and robustness evaluation. **(A)** Multiple-testing screening across 15,728 inverse-variance weighted exposure-outcome tests, showing the reduction from 659 nominal associations to three corrected-significant, taxonomically related findings. **(B)** Forest plot of the corrected associations between genetically predicted genus *Bifidobacterium*, order Bifidobacteriales, and family Bifidobacteriaceae abundance and urinary tract infection risk. Points represent odds ratios, and horizontal lines represent 95% confidence intervals. **(C)** Representative SNP-level MR scatter plot for genus *Bifidobacterium* and urinary tract infection. **(D)** Leave-one-out analysis for genus *Bifidobacterium*, showing that the protective estimate remained directionally consistent after sequential omission of each SNP. **(E)** Sensitivity matrix summarizing instrument strength, Cochran's Q, MR-PRESSO global tests, leave-one-out analysis, Steiger directionality, and MR-Egger intercept results. FDR, false discovery rate; MR, Mendelian randomization; SNP, single-nucleotide polymorphism.

These three results represent a coherent taxonomic hierarchy rather than three independent biological discoveries. Their shared direction and similar effect sizes indicate that the strongest corrected signal in the dataset was a *Bifidobacterium*-related protective association with UTI. Beyond the corrected UTI findings, the nominal associations revealed recurrent patterns across microbial lineages, anatomical sites, and infection categories. Although these observations require further confirmation, they provide an exploratory overview of potential microbiota–infection relationships across the 75 outcomes and may help guide subsequent replication and experimental studies.

## Instrument strength and sensitivity analyses

To further evaluate the reliability of the corrected *Bifidobacterium*-related UTI signals, we assessed instrument strength and performed complementary sensitivity analyses. Instrument-strength calculations did not suggest substantial weak-instrument bias under the conventional *F* > 10 criterion. Across all exposure-outcome analyses, the minimum exposure-level average *F*-statistic was 20.22. For the corrected UTI findings, the mean *F*-statistics were 26.72 for genus *Bifidobacterium* and 27.33 for both order Bifidobacteriales and family Bifidobacteriaceae. The corresponding minimum SNP-level *F*-statistics were 19.00, 15.83, and 15.83, respectively. Cumulative exposure *R*^2^ values were 0.0450 for genus *Bifidobacterium* and 0.0357 for the order and family features (Supplementary Table 2).

The corrected findings were generally supported by complementary sensitivity analyses (Figures 1C–E). Cochran's *Q*-tests did not indicate heterogeneity for genus *Bifidobacterium* (*P* = 0.386), order Bifidobacteriales (*P* = 0.465), or family Bifidobacteriaceae (*P* = 0.465). MR-PRESSO global tests were also not significant (*P* = 0.414, 0.495, and 0.526, respectively), and no outlier-corrected estimate was generated because no outlier was identified. Leave-one-out analyses showed that the protective estimates remained directionally consistent after sequential removal of individual SNPs, suggesting that the associations were not driven by a single influential instrument. Steiger comparisons were also directionally supportive, as the instruments explained more variance in each microbial exposure than in UTI (genus: *R*^2^ exposure 0.0450 vs. *R*^2^ outcome 0.00516; order and family: 0.0357 vs. 0.00475).

Nevertheless, MR-Egger intercept tests were significant for the genus, order, and family-level *Bifidobacterium* features, indicating that potential directional pleiotropy could not be fully excluded. We therefore interpreted these corrected associations as the strongest MR-supported signals in the dataset, rather than as definitive causal estimates. In addition, sensitivity analyses of the nominally significant associations were broadly supportive, with most associations showing no substantial heterogeneity, no MR-PRESSO-detected outliers, and no single-SNP-driven pattern in leave-one-out analyses. These exploratory sensitivity results were used to characterize the overall stability of the nominal association landscape, while the main causal interpretation remained focused on the corrected *Bifidobacterium*-related UTI signals.

Focused reverse MR analyses were attempted for the corrected UTI–*Bifidobacterium*-related findings and selected Coriobacteriia–respiratory infection pairs. Candidate infection-associated instruments were identified, but no SNPs overlapped with the corresponding microbiota GWAS datasets during instrument matching; therefore, reverse-effect estimates could not be calculated (Supplementary Table 3).

## Systematic exploratory landscape across microbial taxa and infectious outcomes

At the nominal significance threshold, 659 exposure–outcome associations were identified across multiple microbial taxa and infectious outcomes (Figures 2, 3). Taxon-frequency analysis showed that family Bifidobacteriaceae, genus *Enterorhabdus*, and family Lachnospiraceae were among the features associated with the largest numbers of infectious phenotypes. Family Bifidobacteriaceae was nominally associated with 11 outcomes across bacterial and viral categories, while genus *Enterorhabdus* and family Lachnospiraceae showed different distributions across infection groups. These findings provided an overview of recurrent microbial features rather than evidence of broadly shared causal effects.

**Figure 2:**
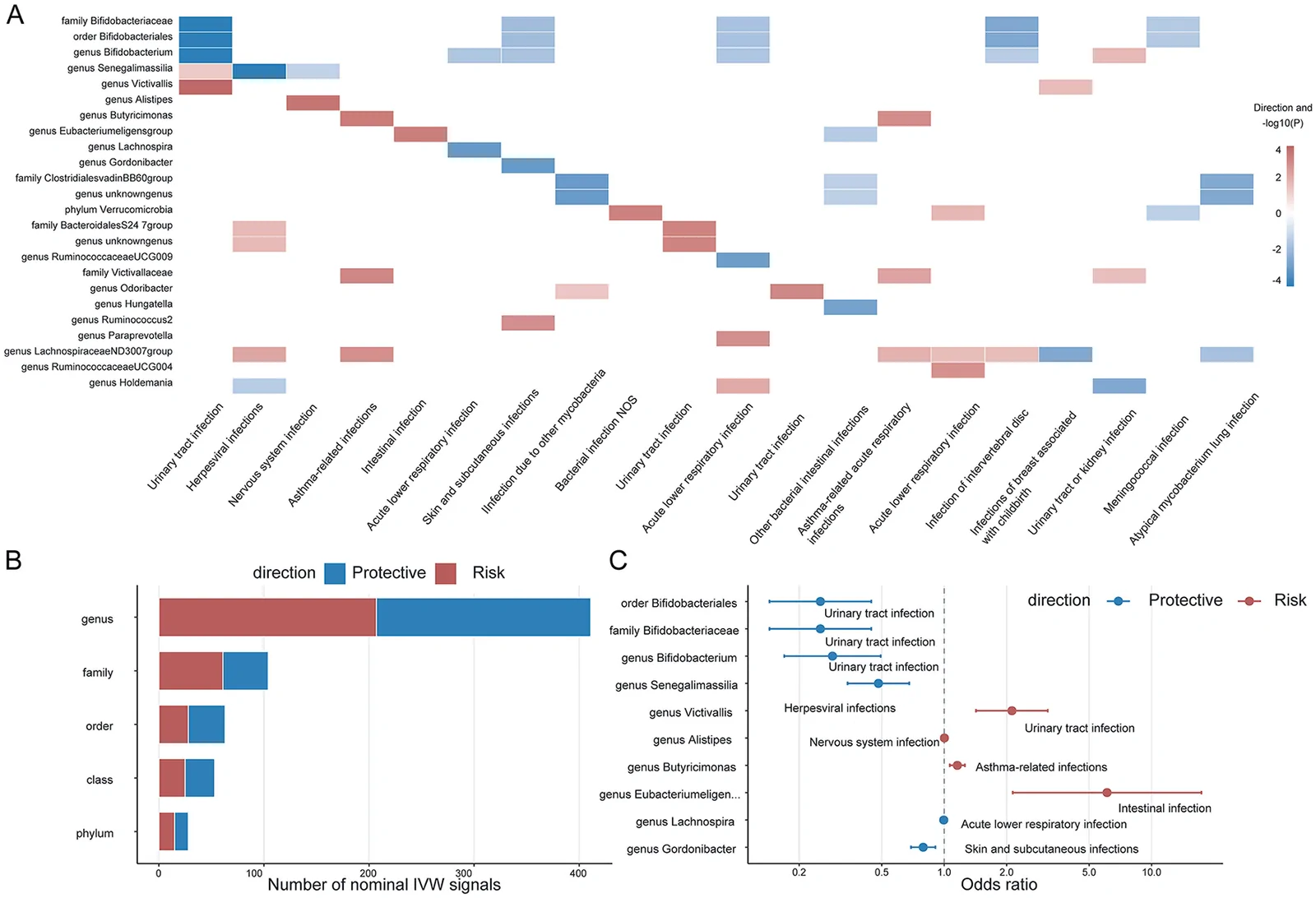
Exploratory landscape of nominal microbiota-infection associations. **(A)** Heatmap of representative nominal inverse-variance weighted associations selected to display the breadth and direction of the microbiota-infection landscape. Red indicates risk-direction estimates and blue indicates protective-direction estimates. **(B)** Distribution of the 659 nominal associations across microbial taxonomic levels. **(C)** Representative exposure-outcome pairs with the largest nominal effect estimates. All panels are hypothesis generating; the displayed associations did not necessarily remain significant after global multiple-testing correction. Complete odds ratios, confidence intervals, nominal *P*-values, adjusted *P*-values, and SNP counts are provided in the Supplementary Tables.

**Figure 3:**
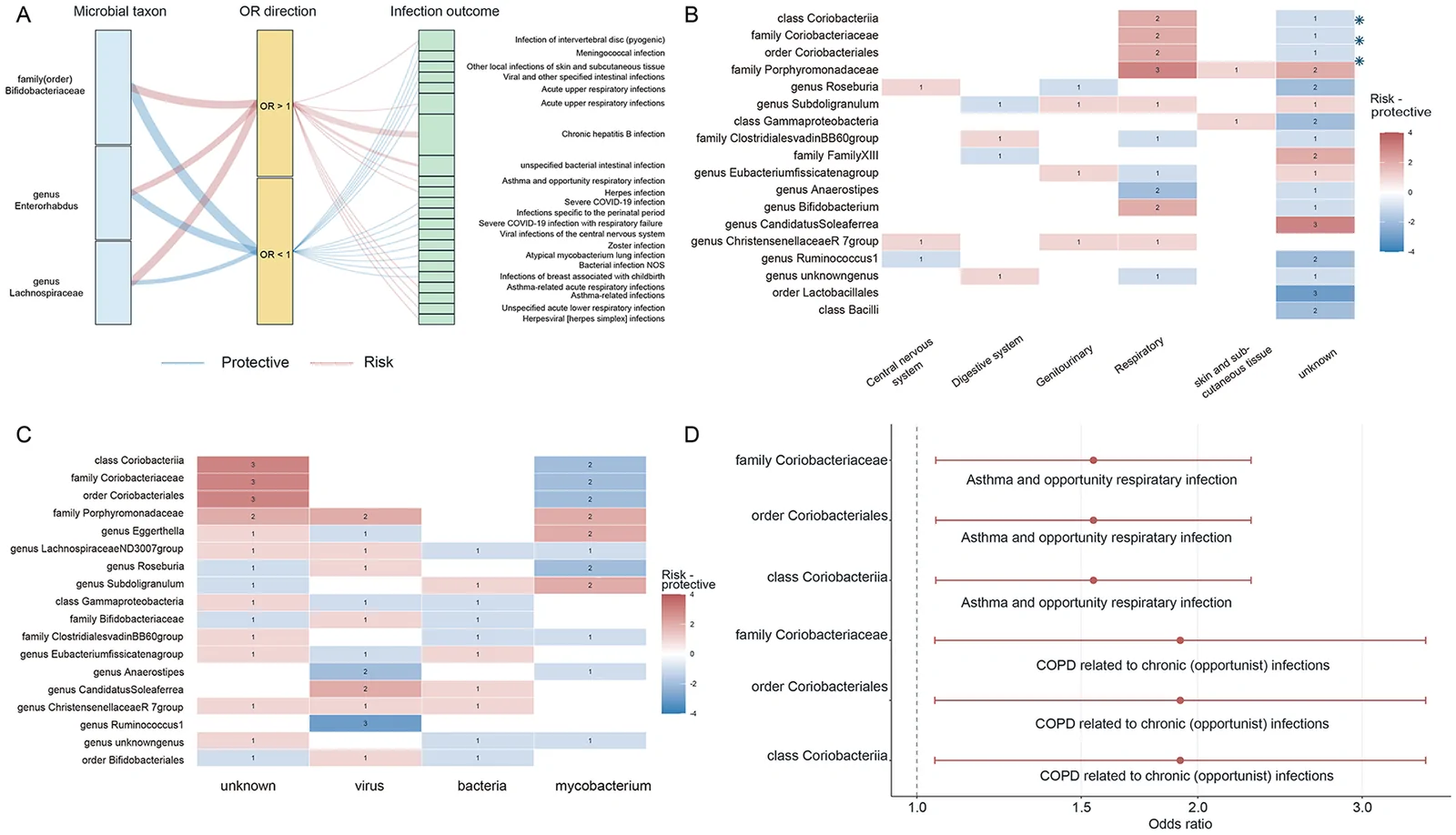
Exploratory infection-specific microbial patterns and follow-up target selection. **(A)** Sankey diagram linking recurrent microbial taxa to the direction of their nominal MR estimates and to associated infectious outcomes. Link width reflects the number of represented nominal associations rather than corrected statistical significance. **(B)** Heatmap of microbial patterns across anatomical infection sites. Rows were selected using the pre-specified strong nominal-signal rule, and the Coriobacteriia lineage selected for experimental follow-up is marked. **(C)** Heatmap comparing microbial patterns across pathogen groups using the same selection rule as in panel **(B)**. **(D)** Forest plot of nominal Coriobacteriia-lineage associations with respiratory outcomes. The repeated class, order, and family-level pattern provided the rationale for selecting *C. aerofaciens* as a representative follow-up species. Panels **(A–D)** are exploratory and should not be interpreted as globally corrected causal discoveries.

When the associations were organized by anatomical site, respiratory outcomes showed the clearest clustering (Figure 3B). Several nominal respiratory associations involved class Coriobacteriia, order Coriobacteriales, and family Coriobacteriaceae, whereas genus *Anaerostipes* appeared among selected protective signals. The recurrence of Coriobacteriia-related features across multiple taxonomic levels provided the rationale for prioritizing this lineage for experimental follow-up.

Pathogen-group and hepatitis-subtype analyses further indicated variation in the distribution of nominal associations (Figure 3C and Supplementary Figure S1). Actinobacteria- and Firmicutes-related features appeared more frequently among viral outcomes, whereas genera *Eggerthella* and *Subdoligranulum* were represented among bacterial outcomes. Different microbial patterns were also observed across acute hepatitis A, chronic hepatitis B, and chronic hepatitis C. Complete taxon-specific effect estimates, confidence intervals, *P*-values, adjusted *P*-values, and SNP counts are provided in the Supplementary Table 1.

## *C. aerofaciens* induced epithelial inflammatory responses

*C. aerofaciens* was selected as an exploratory experimental model because Coriobacteriia-lineage features clustered with respiratory outcomes in the nominal MR landscape and *C. aerofaciens* is a representative species within this lineage (Figures 3D, 4A). In the CCK-8 assay, A549 cell viability declined with increasing concentrations of *C. aerofaciens* culture supernatant, with mean viability of 97.6%, 95.8%, 59.1%, and 17.4% at 10%, 20%, 50%, and undiluted supernatant, respectively, relative to matched controls (Figure 4E).

**Figure 4:**
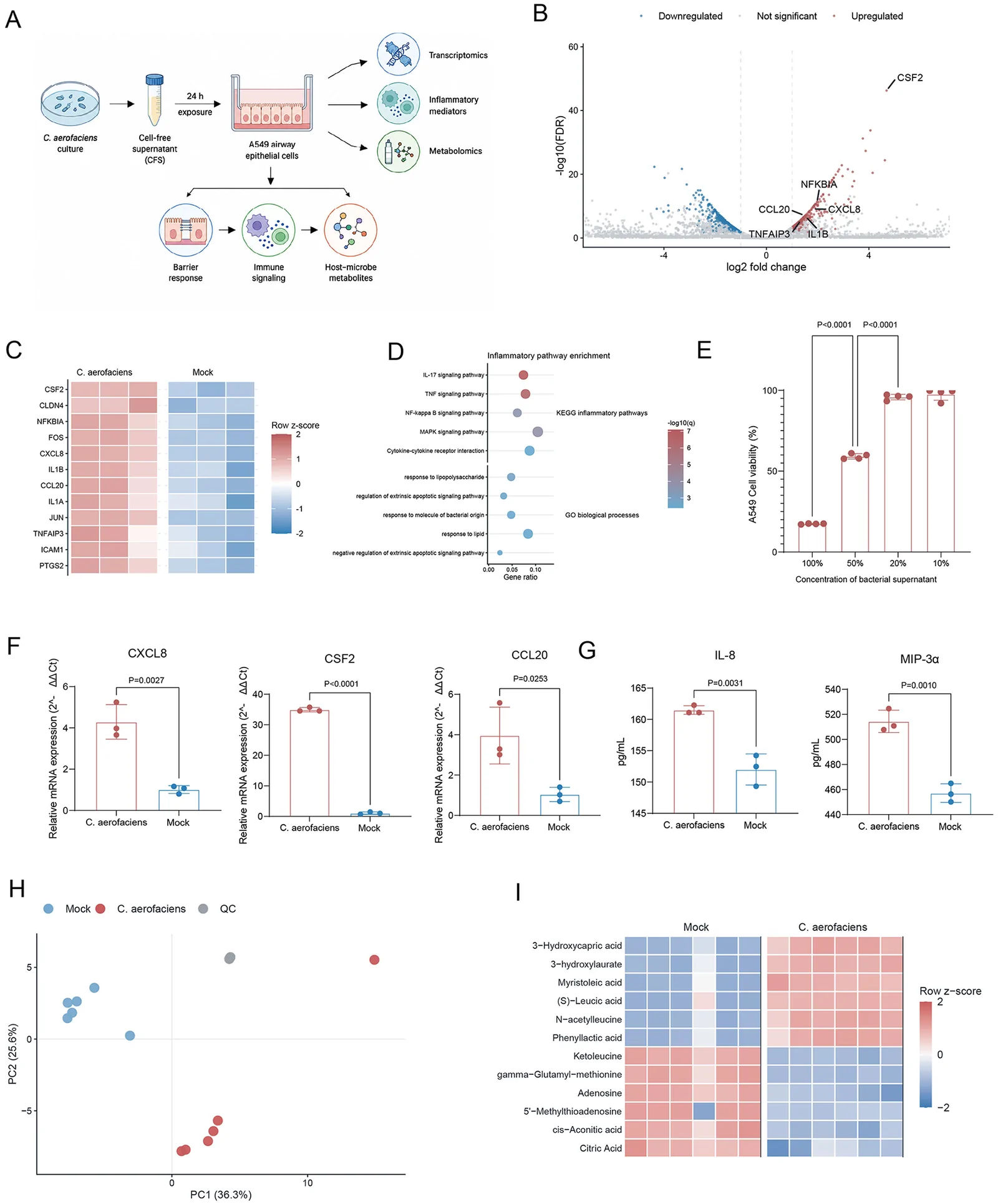
Epithelial and extracellular metabolic responses to *Collinsella aerofaciens* cell-free supernatant. **(A)** Experimental workflow. **(B)** RNA-seq volcano plot. **(C)** Heatmap of selected inflammatory genes. **(D)** GO and KEGG enrichment of differentially expressed genes. **(E)** A549 viability after exposure to different CFS concentrations. **(F)** qPCR validation of CXCL8, CSF2, and CCL20. **(G)** ELISA measurements of IL-8 and MIP-3α; GM-CSF was below the lower limit of quantification and is not shown. **(H)** PCA of extracellular metabolomic profiles. **(I)** Heatmap of selected Level 1 differential metabolites. Panels **(B–D)** included three biological replicates per group. Data in panels **(E–G)** are presented as mean ± SEM from three independent biological replicates. Panel **(E)** was analyzed by one-way ANOVA with Dunnett's test, and panels **(F, G)** by two-sided unpaired Student's *t*-tests; exact *P*-values are shown. Panels **(H, I)** included six samples per group, and heatmap values are row-scaled *z*-scores.

RNA sequencing compared A549 samples treated with *C. aerofaciens* culture supernatant and BHI mock-control samples after 24 h. The CA-vs.-BHI comparison identified 993 differentially expressed genes, including 275 upregulated and 718 downregulated genes (Figures 4B, C and Supplementary Table 5). Upregulated inflammatory transcripts included *CSF2* (log2 fold change 4.69; adjusted *P* = 5.36 x 10^−47^), *CXCL8* (1.76; adjusted *P* = 7.72 x 10^−10^), and *CCL20* (1.52; adjusted *P* = 1.04 x 10^−7^). Enrichment analyses supported involvement of IL-17, TNF, MAPK, NF-kappa B, cytokine-cytokine receptor, response to bacterial-origin molecules, and inflammatory-response pathways (Figure 4D). Targeted validation was consistent with the transcriptomic findings. qPCR confirmed increased expression of selected inflammatory transcripts, including *CSF2, CXCL8*, and *CCL20*, in *C. aerofaciens-*treated A549 cells compared with BHI mock controls (Figure 4F). ELISA measurements also showed increased secretion of IL-8/CXCL8, and CCL20/MIP-3α in the culture medium after treatment, whereas GM-CSF/CSF2 concentrations remained below the assay's lower limit of quantification (Figure 4G). These transcript- and protein-level measurements provided orthogonal support for the inflammatory response observed in RNA sequencing.

Untargeted metabolomic profiling showed an overall separation between six *C. aerofaciens* cell-free supernatant samples and six matched BHI controls, while the quality-control samples clustered closely (Figure 4H). Using VIP ≥ 1, FDR < 0.05, and a fold change ≥ 2 or ≤ 0.5, 201 differential metabolites were identified, including 102 increased and 99 decreased features (Supplementary Table 6). Selected Level 1 metabolites showed increased (S)-leucic acid, myristoleic acid, 3-hydroxylaurate, and phenyllactic acid, together with decreased adenosine, ketoleucine, cis-aconitic acid, and citric acid in the *C. aerofaciens* supernatant (Figure 4I). These changes were consistent with extracellular metabolic remodeling during *C. aerofacien*s growth.

## Discussion

This study provides a systematic assessment of genetically predicted gut microbiota associations across 75 infectious outcomes, followed by focused experimental investigation of an exploratory respiratory pattern. After global multiple-testing correction, three taxonomically related *Bifidobacterium*–urinary tract infection (UTI) associations remained significant. The broader nominal analysis organized additional patterns across infection sites, pathogen groups, and hepatitis subtypes, providing a framework for prioritizing signals for replication and experimental study.

The corrected findings represent a coherent taxonomic hierarchy rather than three independent biological discoveries. Higher genetically predicted abundance of genus *Bifidobacterium*, order Bifidobacteriales, and family Bifidobacteriaceae was associated with lower UTI risk. The direction of this association is consistent with the recognized role of *Bifidobacterium* as a component of the healthy human gut microbiota (Sadeghpour Heravi and Hu, 2023; O'Callaghan and van Sinderen, 2016). *Bifidobacterium*-related features have also been associated with selected infectious or inflammatory phenotypes in genetic studies (Bian et al., 2024), while microbiota composition involving *Bifidobacterium* has been linked to immune regulation and vaccine responses (Kaushal, 2024; Ng et al., 2022). These observations provide biological context for the present UTI finding, although they do not establish that increasing *Bifidobacterium* abundance would prevent UTI.

The broader nominal results require a more context-dependent interpretation. Bifidobacteriaceae-related features appeared in both protective and risk-direction associations across different infectious outcomes, suggesting that the observed effects may vary according to the infection phenotype, taxonomic level, host context, and underlying microbial function. Taxonomic correlations and residual pleiotropy may also contribute to the similar estimates observed across related microbial features. Accordingly, the corrected result is interpreted as a UTI-specific association rather than evidence for a uniform protective effect of *Bifidobacterium* across infections.

The Coriobacteriia-related respiratory pattern was selected for exploratory experimental follow-up because related microbial features repeatedly clustered with respiratory outcomes in the nominal analysis. Previous studies have connected Coriobacteriia-related taxa with inflammatory responses, cytokine profiles, respiratory outcomes, and intestinal barrier markers (Mizutani et al., 2022; Wang et al., 2024; Efremova et al., 2024; Jin et al., 2023; Svensson et al., 2021). *Collinsella aerofaciens* was therefore used as a representative cultivable species to examine whether its bacterial products could induce an epithelial response in an A549 model. This experimental design linked the exploratory MR landscape to a focused cellular question without assuming that the species-level experiment represented all Coriobacteriia taxa or respiratory outcomes.

*C. aerofaciens* cell-free supernatant induced coordinated changes in inflammatory transcripts and was associated with IL-17, TNF, NF-κB, MAPK, and cytokine–receptor pathways, which are relevant to epithelial responses during infection and inflammatory injury (Mcaleer and Kolls, 2014; Bals and Hiemstra, 2004). qPCR supported increased expression of selected targets, and ELISA detected IL-8 and MIP-3α, whereas GM-CSF remained below the assay's lower limit of quantification. Metabolomic profiling additionally showed increased (S)-leucic acid, hydroxylated fatty acids, and phenyllactic acid, together with decreased ketoleucine, adenosine, cis-aconitic acid, and citric acid, indicating concurrent metabolite production and substrate utilization during bacterial growth. These findings support an epithelial inflammatory response to the cell-free supernatant alongside remodeling of the bacterial extracellular metabolome. Although *C. aerofaciens* can produce immunologically active lipid structures (Kwon et al., 2023), the present data do not identify a specific metabolite responsible for the epithelial response.

Other nominal patterns, including *Anaerostipes*-associated protective signals and differences across pathogen groups or hepatitis subtypes, may help prioritize future studies. Such patterns could reflect differences in transmission route, affected tissue, disease duration, treatment exposure, or immune response, but they may also be influenced by phenotype heterogeneity and multiple testing. Their value in the present study is therefore mainly in organizing the association landscape and identifying candidates for independent replication.

Steiger analyses generally supported the pre-specified microbiota-to-infection direction, including for the corrected UTI findings, but they cannot fully exclude reverse causation or disease-related changes in microbial composition. Focused reverse MR analyses were attempted for selected UTI-related and Coriobacteriia–respiratory pairs, but no overlapping SNPs were available after instrument matching, so reverse-effect estimates could not be calculated. The directionality results should therefore be regarded as complementary evidence rather than a replacement for a fully powered reverse MR analysis. Similarly, the supportive *F*-statistics and sensitivity analyses strengthen confidence in instrument relevance, but they do not eliminate the possibility of horizontal pleiotropy.

## Limitations

Several limitations should be considered. The exposure and outcome GWAS datasets were mainly derived from European-ancestry populations, and participant overlap could not be assessed using summary-level data. Differences in ancestry, linkage disequilibrium, phenotype definition, and case ascertainment may affect generalizability. The relatively permissive microbiota instrument threshold may also increase susceptibility to pleiotropy, and current microbiome GWAS data cannot resolve strain-level functions.

The experimental analysis used one bacterial species, one transformed epithelial cell line, one treatment duration, and unfractionated bacterial supernatant. Although transcriptomic, metabolomic, qPCR, and ELISA results provided complementary evidence, metabolite annotations were not confirmed with authentic standards and pathway-inhibition experiments were not performed. Further validation in primary epithelial cells, epithelial–immune co-culture systems, and pathway-specific models will be needed to establish broader biological relevance.

## Methods

## Study design

This study combined a two-sample Mendelian randomization (MR) framework with an exploratory cellular and multi-omics investigation (Figure 5). Genetic associations with gut microbiota (GM) taxa were obtained from the MiBioGen consortium and evaluated against 75 infectious outcomes obtained from publicly available genome-wide association study (GWAS) resources. The MR analyses distinguished associations that remained significant after global multiple-testing correction from nominal associations used for exploratory pattern analysis. A Coriobacteriia-related respiratory pattern identified in the exploratory analysis was subsequently examined using an A549 epithelial cell model and *Collinsella aerofaciens*.

**Figure 5:**
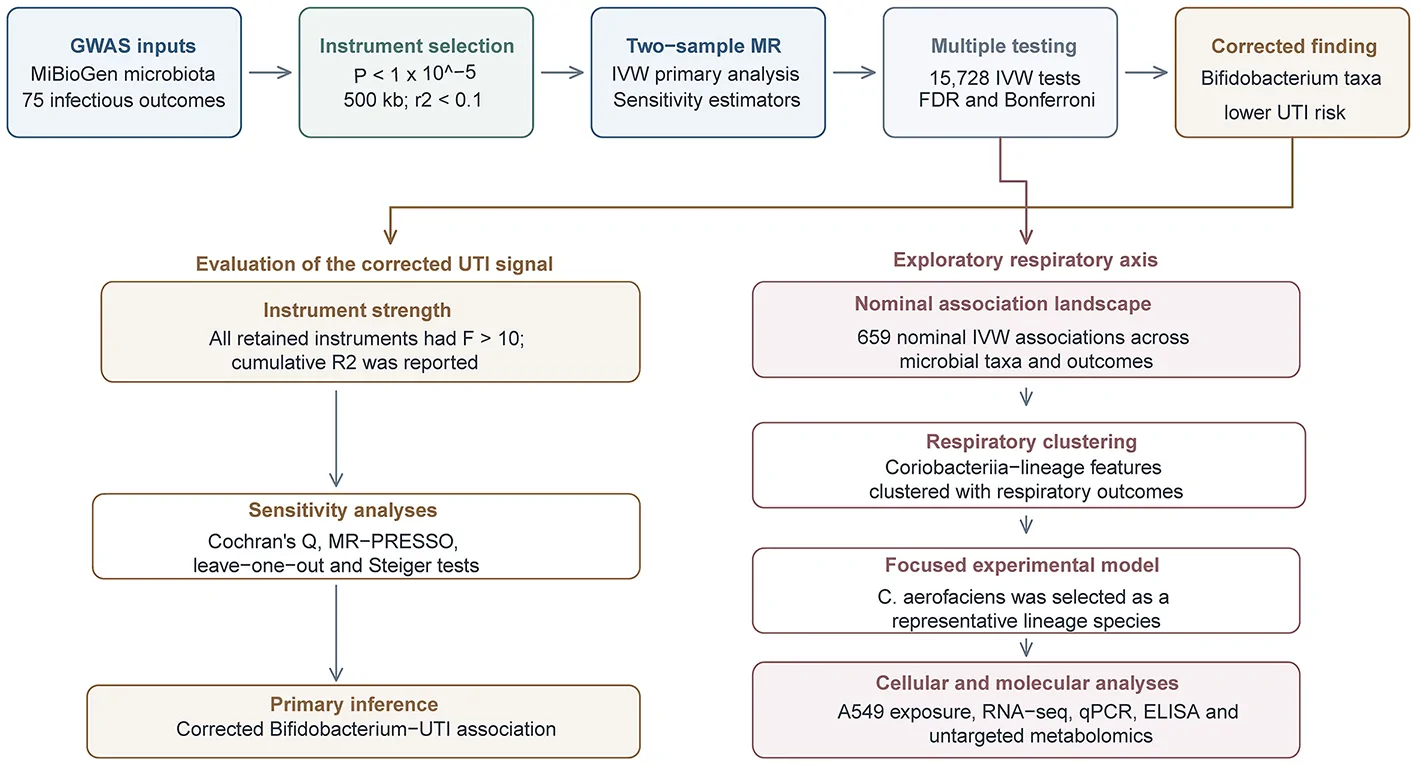
Study design and evidence hierarchy. The study integrated large-scale two-sample Mendelian randomization (MR) with an exploratory epithelial-cell experiment. (A) Gut microbiota genome-wide association study (GWAS) data from the MiBioGen consortium were used as exposures, and 75 infectious outcomes from the GWAS Catalog, IEU OpenGWAS, and FinnGen were used as outcomes. Microbiota-associated single-nucleotide polymorphisms were selected and clumped before exposure-outcome harmonization. (B) Inverse-variance weighted MR was used as the primary analysis. False discovery rate and Bonferroni correction distinguished corrected-significant findings from nominal exploratory associations. (C) Instrument strength, Cochran's Q, MR-Egger intercept, leave-one-out analysis, MR-PRESSO, and Steiger directionality testing were used to evaluate robustness. (D) Nominal associations were organized by taxonomic feature, anatomical infection site, and pathogen group. The recurrent Coriobacteriia-lineage respiratory signal motivated exploratory follow-up using *Collinsella aerofaciens* cell-free supernatant and A549 alveolar epithelial cells.

The MR analyses were based on the three principal instrumental-variable assumptions: the selected genetic variants were associated with the microbial exposure, were independent of major confounders of the exposure-outcome association, and affected the outcome primarily through the microbial feature. Because these assumptions cannot be verified directly using summary-level data, instrument strength, heterogeneity, directional horizontal pleiotropy, outlier-related pleiotropy, single-variant influence, and exposure-to-outcome direction were evaluated using complementary sensitivity analyses.

## GWAS data sources

Genetic associations with GM were obtained from the MiBioGen meta-analysis, which included 18,340 participants from 24 cohorts and was pre-dominantly composed of individuals of European ancestry (*n* = 13,266) (Kurilshikov et al., 2021). The 75 outcome GWAS datasets were downloaded from the GWAS Catalog, IEU OpenGWAS, and FinnGen. Dataset source, phenotype definition, sample size, case and control counts, ancestry, and genome build are summarized in Supplementary Table 1 where the information was available. Because individual-level data and participant identifiers were unavailable, overlap between exposure and outcome cohorts could not be quantified directly.

Candidate outcomes were screened according to the phenotype names and definitions provided by the original GWAS repositories. Outcomes were included when: (1) the phenotype represented a human infectious disease or a clinically defined infection-related condition; (2) publicly accessible summary statistics were available; and (3) the dataset contained the single-nucleotide polymorphism (SNP) identifiers, effect alleles, effect estimates, and standard errors required for harmonization and MR analysis. Duplicate datasets representing the same phenotype and datasets with insufficient variant-level information were excluded, whereas clinically distinct infection subtypes were retained. The final analysis included 75 outcomes covering bacterial, viral, fungal, parasitic, mycobacterial, site-defined, and other infectious phenotypes. Their definitions and data-source characteristics are provided in Supplementary Table 1.

## Instrumental variable (IV) selection

Microbiota-associated SNPs were selected at *P* < 1 x 10^−5^ from the MiBioGen top-hit dataset (Kurilshikov et al., 2021; Van Der Velde et al., 2019). To reduce correlation among instruments, variants were clumped using a 500-kb window and an *r*^2^ threshold of 0.1 according to previous studies (Sanna et al., 2019; Zhang et al., 2024; Ni et al., 2022). The exposure and outcome datasets were harmonized by aligning the effect alleles. SNPs absent from the corresponding outcome dataset were excluded. Palindromic or ambiguous variants that could not be aligned reliably were also removed. Only harmonized SNPs retained for MR analysis were included in subsequent instrument-strength and sensitivity analyses.

Instrument strength was evaluated for each retained SNP as: *F* = β_exposure^2^ / SE_exposure^2^, where β_exposure and SE_exposure represent the SNP-exposure effect estimate and its standard error, respectively. An *F*-statistic >10 was used as the conventional indicator against substantial weak-instrument bias. SNP-level variance explained was estimated using the exposure effect estimate, effect-allele frequency, and exposure sample size. The cumulative *R*^2^ and mean *F*-statistic were subsequently summarized for each exposure-outcome pair.

## Mendelian randomization analysis

The inverse-variance weighted (IVW) estimator was used as the primary MR method. Weighted-median and MR-Egger estimates were calculated as complementary estimators where sufficient genetic instruments were available. MR effect estimates were expressed as odds ratios (ORs) with 95% confidence intervals (CIs) for the infectious outcomes. The weighted-median estimator can provide a consistent estimate when at least half of the total instrument weight is contributed by valid instruments. MR-Egger regression allows the average pleiotropic effect to differ from zero but generally provides less precise estimates. The primary interpretation was therefore based on the IVW results together with the direction, magnitude, and sensitivity profile of each association.

## Multiple-testing correction

Multiple testing was evaluated across all 15,728 IVW exposure-outcome tests. Benjamini-Hochberg false discovery rate (FDR)-adjusted *P*-values were calculated across the complete set of IVW tests. A global Bonferroni significance threshold was additionally calculated as 0.05/15,728 = 3.179 × 10^−6^. Associations meeting FDR < 0.05 were considered significant after control of the expected false-discovery proportion. Associations with *P* < 3.179 × 10^−6^ additionally met the global Bonferroni threshold. Associations with nominal IVW *P* < 0.05 that did not meet either global correction criterion were retained as exploratory findings and were not interpreted as independently confirmed causal associations.

## Sensitivity analyses

Cochran's Q statistic was used to evaluate heterogeneity among SNP-specific causal estimates. Directional horizontal pleiotropy was assessed using the MR-Egger intercept test. A statistically significant intercept was interpreted as evidence that directional pleiotropy could not be excluded. Leave-one-out analysis was performed by removing each SNP sequentially and recalculating the IVW estimate. This analysis evaluated whether an association was disproportionately influenced by a single genetic variant. The MR-PRESSO global test was used to assess evidence of outlier-related horizontal pleiotropy when the number of available instruments was sufficient. When no outlier was detected, an outlier-corrected estimate was not generated. MR-PRESSO was run using 3,000 simulation distributions and a significance threshold of 0.05. Steiger directionality analysis compared the variance explained by the instruments in each microbial exposure and infectious outcome. Directionality was supported when the instruments explained more variance in the exposure than in the outcome. Sensitivity analyses were performed for the multiple-testing-corrected findings and, where the required number of SNPs and input variables were available, for the nominally significant IVW associations.

## Exploratory organization of nominal associations

The 659 nominally significant IVW associations (*P* < 0.05) were grouped by anatomical infection site and pathogen category. For the heatmaps, associations with OR > 1.5 or OR < 0.5 were defined as strong risk-enhancing or protective signals, respectively. Taxa were ranked by their aggregate directional scores, and the top 18 were displayed, with Coriobacteriia-lineage taxa retained to illustrate the selection of the experimental model. A descriptive Sankey diagram summarized the relationships among selected microbial taxa, effect directions, and infectious outcomes. The focused analysis included all nominally significant Coriobacteriia-respiratory associations.

## Experimental design and sample collection

*C. aerofaciens* (ATCC 25986) was selected as a representative species within the Coriobacteriia lineage because several lineage-level microbial features clustered with respiratory infection outcomes in the exploratory MR analysis. The bacterium was cultured in Brain Heart Infusion (BHI) broth under strictly anaerobic conditions at 37 °C. At the late logarithmic growth phase, cultures were centrifuged at 4,000 × g for 10 min at 4 °C, and the supernatant was filtered through a 0.22-μm sterile filter to obtain cell-free supernatant (CFS). Uninoculated BHI medium was processed identically and used as the mock control. Aliquots of the CFS and BHI control were subjected to untargeted metabolomic profiling.

A549 human alveolar epithelial cells were maintained in DMEM supplemented with 10% fetal bovine serum and 1% penicillin-streptomycin at 37 °C in a humidified atmosphere containing 5% CO_2_. Cells were seeded and allowed to adhere overnight before treatment with *C. aerofaciens* CFS or the matched BHI control for 24 h. Cells were collected for RNA sequencing, and for qPCR validation of *CSF2, CXCL8*, and *CCL20*, using *GAPDH* as the reference gene. Conditioned culture media were collected for ELISA measurement of GM-CSF, IL-8, and MIP-3α. Cell viability was evaluated in parallel CCK-8 experiments using different concentrations of CFS.

## Cell viability assay

Cell viability was evaluated using the Cell Counting Kit-8 (CCK-8) assay (YEASEN, Cat. 40203ES60) according to the manufacturer's instructions. Briefly, A549 cells were seeded in 96-well plates at a density of 1 × 10^4^ cells/well. Following overnight incubation, cells were treated with various concentrations of *C. aerofaciens* (10%, 20%, 50%, 100% v/v) CFS or an equivalent volume of mock BHI medium for 24 h. Subsequently, 10 μl of CCK-8 solution was added to each well, and the plates were incubated for an additional 2 h at 37 °C. The absorbance was measured at 450 nm using a microplate reader.

## RNA sequencing and transcriptomic analysis

RNA sequencing was performed using three independent biological replicates per group. Poly(A)-positive mRNA was enriched using oligo(dT) beads, and sequencing libraries were prepared using the Illumina TruSeq RNA Sample Preparation Kit. The libraries were sequenced on an Illumina NovaSeq 6000 platform using a 150-bp paired-end strategy (PE150). Raw reads were filtered using Trimmomatic v0.39 and aligned to the human GRCh38 reference genome (GCF_000001405.40) using HISAT2 v2.2.1. Gene-level reads were quantified using featureCounts v2.0.6, and differential expression was analyzed using edgeR with |log2 fold change| ≥ 1 and FDR ≤ 0.05. A total of 44.54–50.45 million clean reads were retained per sample. Q30 values ranged from 98.21% to 98.42%, GC content ranged from 43.99% to 44.89%, and mapping rates ranged from 97.66% to 98.14%. GO and KEGG enrichment analyses were conducted using GOATOOLS v0.9.9 and KOBAS v3, respectively. The raw RNA-seq data have been deposited in the Genome Sequence Archive for Human under accession HRA019745 (BioProject PRJCA069456).

## Quantitative real-time PCR

qPCR was used to validate RNA-seq-selected inflammatory genes (*CSF2, CXCL8, CCL20*). Total RNA was extracted from independent A549 cell cultures using the RNeasy Mini Kit (Qiagen, Cat. 74104), and first-strand cDNA was synthesized from 1 μg of total RNA using PrimeScript RT Master Mix (Perfect Real Time; Takara Bio, Cat. RR036A). Each 20 μl qPCR reaction contained 20 ng cDNA and TB Green Premix Taq II, and was run on an ABI 7500 Real-Time PCR System (Applied Biosystems) under the following conditions: 95 °C for 30 s, then 40 cycles of 95 °C for 3 s and 60 °C for 30 s, followed by melt curve analysis. Relative expression was calculated using the 2^−Δ*ΔCt*^ method. Primer sequences are provided in Supplementary Table 4. Each group included three independent biological replicates, with three technical qPCR replicates per sample.

## Enzyme-linked immunosorbent assay

After 24 h of treatment with *C. aerofaciens* CFS or the matched BHI control, A549 culture supernatants were collected and centrifuged to remove cellular debris. Secreted GM-CSF/CSF2, IL-8/CXCL8, and MIP-3α/CCL20 were measured using human ELISA kits from Elabscience (Wuhan, China): GM-CSF (Cat. E-EL-H0081), IL-8 (Cat. E-EL-H6008), and MIP-3α (Cat. E-EL-H0027). Assays were performed according to the manufacturer's instructions. Cytokine concentrations were calculated from the corresponding standard curves, corrected for sample dilution, and reported in pg/mL. Each group included three independent biological replicates.

## Untargeted metabolomic analysis

Untargeted metabolomic profiling was performed on six independent *C. aerofaciens* cell-free supernatant samples and six matched BHI controls. Each sample (1 mL) was freeze-dried, resuspended in pre-chilled 80% methanol, incubated on ice for 5 min, and centrifuged at 15,000 × *g* for 15 min at 4 °C. The supernatant was diluted with LC-MS-grade water to a final methanol concentration of 53% and centrifuged again. A pooled quality-control sample was prepared by combining equal aliquots of the study samples and was analyzed periodically to monitor analytical stability. Samples were analyzed by BIOZERON Co., Ltd. using a Vanquish UHPLC system coupled to a Q Exactive HF-series Orbitrap mass spectrometer with positive- and negative-ionization modes.

Raw data were processed using Compound Discoverer 3.3 for peak alignment, detection, and relative quantification. Features with a coefficient of variation >30% in quality-control samples were excluded. Metabolites were annotated against the mzCloud, mzVault, MassList, KEGG, HMDB, and LIPID MAPS databases. PCA and OPLS-DA were performed using MetaboAnalystR, and differential metabolites were defined by VIP ≥ 1, FDR < 0.05, and a fold change ≥ 2 or ≤ 0.5. Selected Level 1 differential metabolites were visualized after log2 transformation and row-wise z-score scaling. Metabolite identities were not independently confirmed using authentic standards.

## Statistical analysis

MR analyses were conducted in *R* version 4.2.1 using TwoSampleMR version 0.6.14 and MR-PRESSO version 1.0 (Burgess and Thompson, 2017; Verbanck et al., 2018). CCK-8, qPCR, and ELISA data were obtained from three independent biological replicates and are presented as mean ± standard error of the mean (SEM). Technical replicates, where applicable, were averaged before statistical analysis. Two-group comparisons were performed using two-sided unpaired *t*-tests. For CCK-8 experiments involving multiple CFS concentrations, one-way analysis of variance followed by Dunnett's multiple-comparison test was used against the matched BHI control. Unless otherwise specified, *P* < 0.05 was considered statistically significant. Multiple-testing correction for transcriptomic and metabolomic analyses was performed as described above.

## Data Availability

The raw RNA-seq data generated in this study have been deposited in the Genome Sequence Archive for Human at the National Genomics Data Center, China National Center for Bioinformation, under accession HRA019745 (BioProject PRJCA069456) and are publicly available. The raw untargeted LC-MS metabolomics data have been deposited in OMIX under accession OMIX019176 (BioProject PRJCA069456) and are publicly available. The GWAS summary statistics analyzed in this study are publicly available from MiBioGen, IEU OpenGWAS, the GWAS Catalog, and FinnGen.
